# Photocatalytic Degradation of 4-Nitrophenol by C, N-TiO_2_: Degradation Efficiency vs. Embryonic Toxicity of the Resulting Compounds

**DOI:** 10.3389/fchem.2018.00192

**Published:** 2018-06-04

**Authors:** Oluwatomiwa A. Osin, Tianyu Yu, Xiaoming Cai, Yue Jiang, Guotao Peng, Xiaomei Cheng, Ruibin Li, Yao Qin, Sijie Lin

**Affiliations:** ^1^College of Environmental Science and Engineering, State Key Laboratory of Pollution Control and Resource Reuse, Shanghai Institute of Pollution Control and Ecological Security, Biomedical Multidisciplinary Innovation Research Institute, Shanghai East Hospital, Tongji University, Shanghai, China; ^2^UN Environment-Tongji Institute of Environment for Sustainable Development, Tongji University, Shanghai, China; ^3^Center for Genetic Epidemiology and Genomics, School of Public Health, Jiangsu Key Laboratory of Preventive and Translational Medicine for Geriatric Diseases, Medical College of Soochow University, Suzhou, China; ^4^Institute for Translational Nanomedicine, Shanghai East Hospital, Institute for Biomedical Engineering & Nano Science, Tongji University School of Medicine, Shanghai, China; ^5^School for Radiological and Interdisciplinary Sciences (RAD-X), Jiangsu Provincial Key Laboratory of Radiation Medicine and Protection Medical College of Soochow University, Suzhou, China

**Keywords:** 4-nitrophenol, mesoporous C, N-TiO_2_, photocatalytic degradation, intermediate compounds, embryonic toxicity

## Abstract

The photocatalytic activity of TiO_2_ based photocatalysts can be improved by structural modification and elemental doping. In this study, through rational design, one type of carbon and nitrogen co-doped TiO_2_ (C, N-TiO_2_) photocatalyst with mesoporous structure was synthesized with improved photocatalytic activity in degrading 4-nitrophenol under simulated sunlight irradiation. The photocatalytic degradation efficiency of the C, N-TiO_2_ was much higher than the anatase TiO_2_ (A-TiO_2_) based on absorbance and HPLC analyses. Moreover, using zebrafish embryos, we showed that the intermediate degradation compounds generated by photocatalytic degradation of 4-nitrophenol had higher toxicity than the parent compound. A repeated degradation process was necessary to render complete degradation and non-toxicity to the zebrafish embryos. Our results demonstrated the importance of evaluating the photocatalytic degradation efficiency in conjunction with the toxicity assessment of the degradation compounds.

## Introduction

Photocatalytic degradation of organic pollutants is considered as an efficient, clean, and cost-effective alternative for contaminated water treatment (Wang et al., 1999; Kiros et al., 2013; Osin et al., 2017). Among various types of nano-photocatalysts, titanium dioxide (TiO_2_) based photocatalysts are the most popular ones owing to their low cost, simple but reliable synthesis methods, resistance to photo-corrosion, and chemical stability (Carp et al., 2004; Herrmann et al., 2007; Rajeshwar et al., 2008; Akpan and Hameed, 2009). TiO_2_ has been used to perform photocatalytic oxidation of contaminants in both aqueous and air media (Fox and Dulay, 1993; Hoffmann et al., 1995; Linsebigler et al., 1995). However, due to their large bandgap (3.2 eV), TiO_2_ requires UV light activation (<387 nm), which means only a small portion of the solar spectrum could be used for photocatalytic applications (Wen et al., 2011).

To increase the utility of the visible light spectrum, one common strategy to improve the efficiency of TiO_2_ based photocatalysts is to dope or co-dope metal or non-metal elements into the crystalline structure (Asahi et al., 2001). Doping with non-metals, such as nitrogen (Asahi et al., 2001; Burda et al., 2003; Diwald et al., 2004; Cheng et al., 2012), carbon (Lettmann et al., 2001; Sakthivel and Kisch, 2003; Dong et al., 2011), fluorine (Li et al., 2005a,b), and sulfur (Ohno et al., 2004; Yu et al., 2005) has proven to be effective to narrow the bandgap. Among them, nitrogen-doped TiO_2_ (N-TiO_2_) resulted a considerable increase of the photocatalytic activity in the visible light spectrum. Carbon doping can make TiO_2_ sensitive to visible light with improved adsorption capacity for organic pollutant molecules (Park et al., 2006; Dong et al., 2011). Moreover, the simultaneous doping of two types of atoms into TiO_2_, such as N–F (Li et al., 2005a,b; Giannakas et al., 2013; Wang et al., 2014), N–S (Yu et al., 2006), and C–N (Wang and Lim, 2010, 2011; Hassan et al., 2014; Trevisan et al., 2014), has recently attracted considerable interests, for the possible integrated or synergistic effects by the co-doped elements.

Meanwhile, it is important to note that a complete degradation of organic pollutants by photocatalysts was difficult to achieve (Dong et al., 2015). And the intermediate compounds generated during photocatalytic degradation might render higher toxicity than their parent compound (Dong et al., 2015; Li et al., 2016). Li et al. previously reported that transformation products (TPs) of acesulfame generated by the photocatalytic processes using TiO_2_ photocatalyst were even more toxic than the parent compound (Li et al., 2016). These results were similar to a study carried out by Sang et al. reporting the successful and substantial photocatalytic degradation of acesulfame and sucralose under simulated natural UV conditions. However, real-time observation revealed that the transformation of acesulfame led to a collection of more persistent by-products that were 500 times more toxic than the parent compound inducing significantly elevated toxicity in both marine bacteria and zebrafish embryos (Sang et al., 2014). Therefore, while evaluating the effectiveness of photocatalytic degradation, it is also important to consider the potential hazardous effects exerted by the intermediate degradation compounds (Li et al., 2017; Osin et al., 2017).

Against this background, we set out to explore the use of C and N co-doping to create a TiO_2_ based photocatalyst with higher catalytic and degradation efficiency toward 4-nitrophenol, a representative organic pollutant. Under simulated sunlight irradiation, the as-synthesized C, N-TiO_2_ showed much higher catalytic activity in comparison with the anatase TiO_2_ (A-TiO_2_) Using model organism zebrafish embryos, we showed the intermediate compounds of 4-nitrophenol were more toxic than the parent compound. And repeated photocatalytic degradation process could render non-toxicity as a result of a complete degradation of 4-nitrophenol.

## Materials and methods

### Materials and reagents

In this study, different concentrations of reagents (Table 1) were used to obtain the best doping element ratios and concentrations, which determined the catalytic activity of the as-synthesized particles. 4-nitrophenol of 98% purity was used as a representing pollutant in this study. In the preparation of the modified TiO_2_, titanium tetrachloride (TiCl_4_, 99.9%), ammonia (NH3.H_2_O, 28–30%) and citric acid (C_6_H_8_O_7_, 95.5%) were used as titanium (Ti), nitrogen (N) and carbon (C) source. Perchloric acid (HClO_4_, 70–72%) was added to serve as a pore-making agent during the synthesis process based on Equation 1:

(1) $$4HClO_{4}\;\to 2H_{2}O\;+7O_{2}\underset{↑}{\sim 300^{{}^{\circ}}C}+\;2Cl_{2}\underset{↑}{\sim 300^{{}^{\circ}}C}$$

All reagents were purchased from Aladdin Reagent Co., Shanghai, China and were of analytical (AR) grade, used without further purification.

**Table 1:** Concentration variations of reagents.

**Parameters**	**Concentration**				
Ammonia (ml)	60	90^*^	120		
Citric Acid (g)	1.15	2.30^*^	3.45	4.61	9.22
Perchloric Acid (ml)	0.7^*^	0.9	1.2		

* *Optimum concentrations which produced the best activity and selectivity of the catalyst*.

### Synthesis and physicochemical characterizations of C, N-TiO_2_

The C, N-TiO_2_ was synthesized by adding TiCl_4_ carefully into citric acid solution and stirred for 30 min. A white suspension was obtained after ammonia (NH_3_) solution was added into the solution dropwise. The mixed solution was stirred for another 30 min in an ice bath. After HClO_4_ was added, the reaction temperature was increased to 100°C while stirring. The slurry obtained was dried in a vacuum oven for 5 h. Thereafter, the light yellow powder was collected and then annealed at 450°C for 2 h with a ramp of 5°C /min in air. The powder obtained was grinded and used for the further analyses.

The size and crystallinity of the as-synthesized C, N-TiO_2_ were determined by transmission electron microscope (JEM-2100, JEOL Ltd., Japan) and X-ray diffractometer (Bruker D8 Advanced XRD, Bruker Co., Germany), respectively. Chemical compositions and oxidation states of the sample were analyzed using X-ray photoelectron spectroscopy (XPS) with monochromated Al Kα radiation (hν = 1486.6 eV). Binding energies were calibrated using C 1s peak of C-C bond set at 284.8 eV. The fitting and analysis of the spectra was performed in XPS PEAK version 4.1. N_2_ adsorption-desorption isotherms were measured on Micromeritics ASAP 2,460 device at 77K. Before detection, the sample was degassed at 373K.

### Photocatalytic degradation

The photocatalytic activities of the C, N-TiO_2_ and commercial anatase-TiO_2_ (A-TiO_2_) were carried out in a photoreactor coupled with a simulated sunlight source (Shanghai Deyangyibang Instruments CO., LTD, DY-D type, also shown in Figure S1). Amount of 20 mL of 4-nitrophenol at 7.0 × 10^−2^ mM was transferred into glass tubes and placed orderly in the photo-reactor. Amount of 10 mg of the C, N-TiO_2_ and A-TiO_2_ were added, and the adsorption process of all experiments was done in the dark for 2 h. A magnetic stirrer was used to achieve a satisfactory suspension of the photocatalyst and the homogeneity of the reacting mixture. Samples at 30 min intervals were withdrawn, centrifuged at 8,000 rpm for 10 min, and used for further analysis. The percent of 4-nitrophenol removal was analyzed using a UV-vis spectroscopy, measuring the absorbance at 317nm, while observed first-order rate constants (*k*, min^−1^) were measured from Equations (2) and (3), respectively.

(2) $$\begin{array}{r} 4-nitrophenol\;removal\;\left(\%\right)=\frac{co-ct}{co}\;\times \;100 \end{array}$$

(3) $$\begin{array}{r} ln\left(\frac{co}{ct}\right)=kt \end{array}$$

Where, Co and Ct are the initial and remaining 4-nitrophenol concentrations at a given time of the reaction; *k* is the observed first-order rate constants.

The degradation efficiency of 4-nitrophenol were also measured by high performance liquid chromatography (HPLC). HPLC analysis was performed on an Agilent 1,100 Series LC system (Agilent technologies, Santa Clara, CA) equipped with a binary pump and an ultraviolet-visible diode array detector to quantify the degradation. Samples were separated on an Eclipse Plus C18 reversed phase HPLC column (2.1 × 150 mm, 100Å, 3.5 μm, Agilent technologies, Santa Clara, CA) with an isocratic gradient of methanol and 5 mM phosphoric acid solution (35:65, v/v) over 15 min at a flow rate of 200 μL/min. Injection volumes were 5 μL and the detection wavelength was 254 nm. The mineralization efficiency was measured by a total organic carbon analyzer (TOC-L-CPH Shimadzu, Japan).

### Zebrafish embryo toxicity testing

The AB wild-type adult zebrafish (*Danio rerio*) were maintained at 28 ± 0.5°C on a 14 h:10 h light/dark cycle in a fish breeding circulatory system (Haisheng, Shanghai, China) and were fed twice daily with live brine shrimps (*Artemia salina*). Two pairs of male/female fish were placed in a single mating box separated by a divider 1 day prior to spawning. Spawning was triggered by removing the divider in the morning and the embryos were collected 2 h afterwards. Using a stereomicroscope (Olympus-SZ61, Olympus Ltd., Japan), healthy and fertilized embryos at 4 h post fertilization (hpf) were selected and placed in U-bottom 96-well plates (Costar-3599, Corning, US), with one embryo per well. Each well was then filled with 200 μL of each concentration series of the test-sample suspension (4-nitrophenol, C, N-TiO_2_, A-TiO_2_ and the degradation products) as well as H-buffer as negative controls. Three replicates were carried out for each treatment, each using 12 embryos. The developmental status of the zebrafish embryos was observed at 24, 48, and 72 hpf. The toxicological endpoints included hatching interference, phenotypic abnormalities and mortality (necrosis of the embryos). All experiments were carried out in accordance with the Animal Ethics Committee at Tongji University, with protocol approved by the Animal Center of Tongji University (Protocol #TJLAC-018-020).

#### Statistical analysis

All treatments were performed with at least three replicates. Data were reported as average ± standard deviations. Statistical analysis was carried out by Student's *t*-test to evaluate the statistical significant differences of the hatching success rate and mortality rate between the treatment groups and the negative control group. *P* < 0.01 was considered statistically significant between experimental and control groups.

## Results and discussion

### Composition and chemical state analysis of C, N-TiO_2_

**Figure 1:**
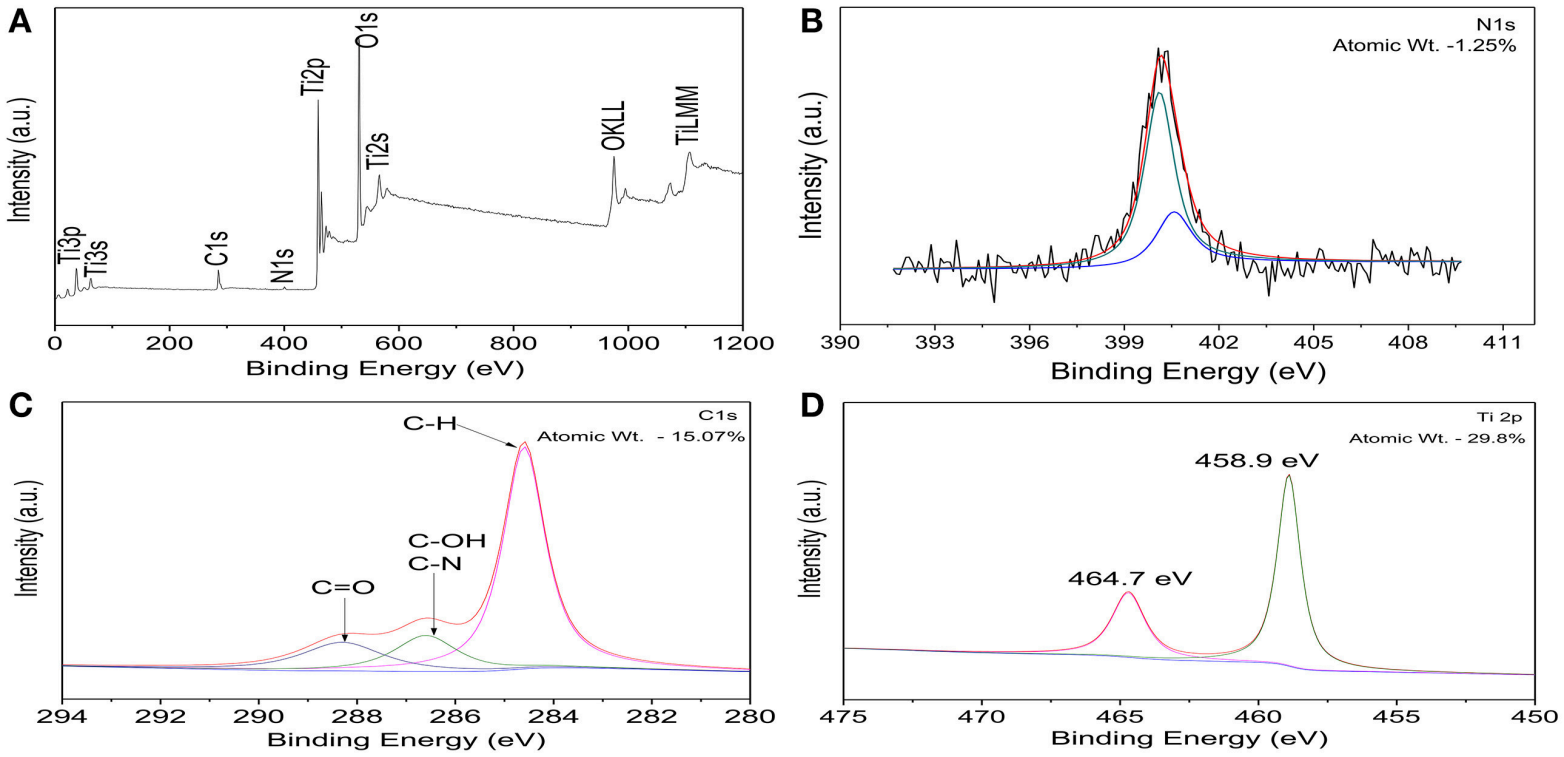
**(A)** XPS survey spectrum of C, N-TiO_2_. High resolution XPS spectra of C, N-TiO_2_: **(B)** N1s **(C)** C1s **(D)** Ti2p.

Figure 1A shows the XPS survey spectrum of C, N-TiO_2_. It was clear that only It, O, N and C elements were detected and the corresponding atomic proportions were 29.8, 53.88, 1.25, and 15.07%, respectively. N and C were assigned to the doping species and adventitious carbon of the apparatus, respectively. The high-resolution XPS spectra of Ti2p, C1s, N1s, and O1s region were shown in Figures 1B–D and Figure S2.

Figure 1B shows the nitrogen region of the XPS measured spectrum for the C, N-doped TiO_2_. The N1s binding energy peaks were broad and asymmetric in the range of 396–403 eV and centered around 400eV (further divided into two peaks centered at 400.1 and 400.5 eV) which can be attributed to the 1 s electron binding energy of the N atom in the environment of O-Ti-N and N-O-Ti (Chen and Burda, 2004; Cong et al., 2007; Xu et al., 2008; Bellardita et al., 2009). Therefore, the nitrogen doped into TiO_2_ mainly existed in the form of substitutional N which resulted in the formation of new energy levels in the forbidden band of TiO_2_ and led to the enhancement of photocatalytic activity in the visible range (Cheng et al., 2012).

Deconvolution of C1s region showed a main peak at 284.6 eV corresponding to C-H and can be assigned to the adventitious carbon contamination adsorbed from the ambient (Wu and Wang, 2013) (Figure 1C). In addition, the peak observed at 284.6 eV refer to the formation of C–OH and C–N bonds. Consequently, the overlap of the peaks corresponding to both C–OH and C–N bonds allows no distinction between the two ones. However, the peak at 288.6 eV indicates the formation of C = O bond that corresponds to a carbonate species present in C-doped titanium systems and that carbon may substitute some of the lattice titanium atoms forming a Ti–O–C structure (Ren et al., 2007). It has been reported that high amount of carbon led to an increased absorption in the visible region, however, it also enhanced the recombination of the charge carriers. This problem was solved by the synergistic effect from the presence of two (or more) complementary dopants (Ren et al., 2007; Li et al., 2010), leading to an improved photoactivity of TiO_2_ in the visible light region, compared to either pure or single-doped TiO_2_.

In the high resolution spectra of Ti_2p_ spectra shown in Figure 1D, peaks at 459.2 and 465 eV corresponds to the spin orbit coupling of Ti2P_3/2_ and Ti2P_1/2_, respectively. It also shows that only the signals corresponding to Ti^4+^ were detected and can be attributed to the binding energy separation between the 2p_1/2_ and 2p_3/2_ peaks of ~5.8 eV (Abdullah et al., 2016). Furthermore, the Ti_2p_ XPS peak of un-doped TiO_2_ has been reported to appear normally at 459.5 eV (Saha and Tomkins, 1992). Therefore, the nitrogen incorporation shifted the XPS spectrum to a lower binding energy (459.2 instead of 459.5 eV). In correspondence with that of N 1s, the O 1s XPS spectra (shown in Figure S2) also shows a broadening at 531.5 eV which confirms the presence of another type of oxygen due to the more covalent nature of N-TiO_2_ (Chi et al., 2007). This might be related to the presence of oxygen and nitrogen from the same lattice units in TiO_2_, which confirmed the interstitial doped form (Cong et al., 2007; Pelaez et al., 2010).

### Phase structure, crystal structure and morphologies of C, N-TiO_2_

**Figure 2:**
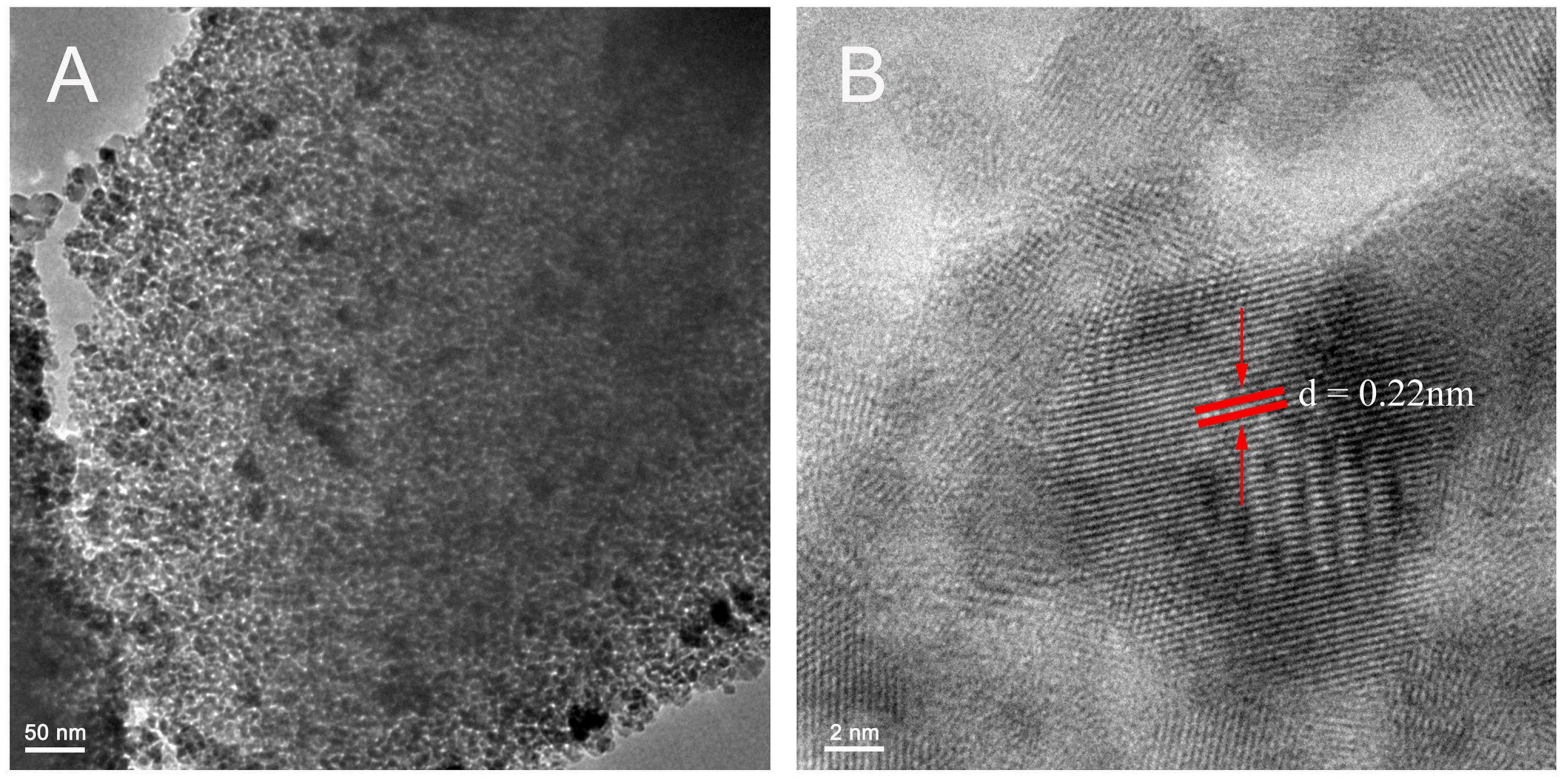
**(A)** Representative TEM image (low magnification) for as-prepared C, N-TiO_2_. **(B)** Representative TEM image (high magnification) for as-prepared C, N-TiO_2_. The distance between the lattice fringes (red parallel lines) pointed by the red arrows is 0.22 nm.

The representative TEM images in Figure 2 illustrate the crystal phase as well as the size distribution of C, N-TiO_2_. The product showed an obvious mesoporous structure caused by the aggregation of C, N-TiO_2_ nanograins of 10–20 nm in size, which were also shown in the SEM image of Figure S3. HClO_4_ was a novel pore-making agent in our recipe of preparing C, N-TiO_2_. As shown in Figure S4, without the addition of HClO_4_, the product looks amorphous with very few pores.

**Figure 3:**
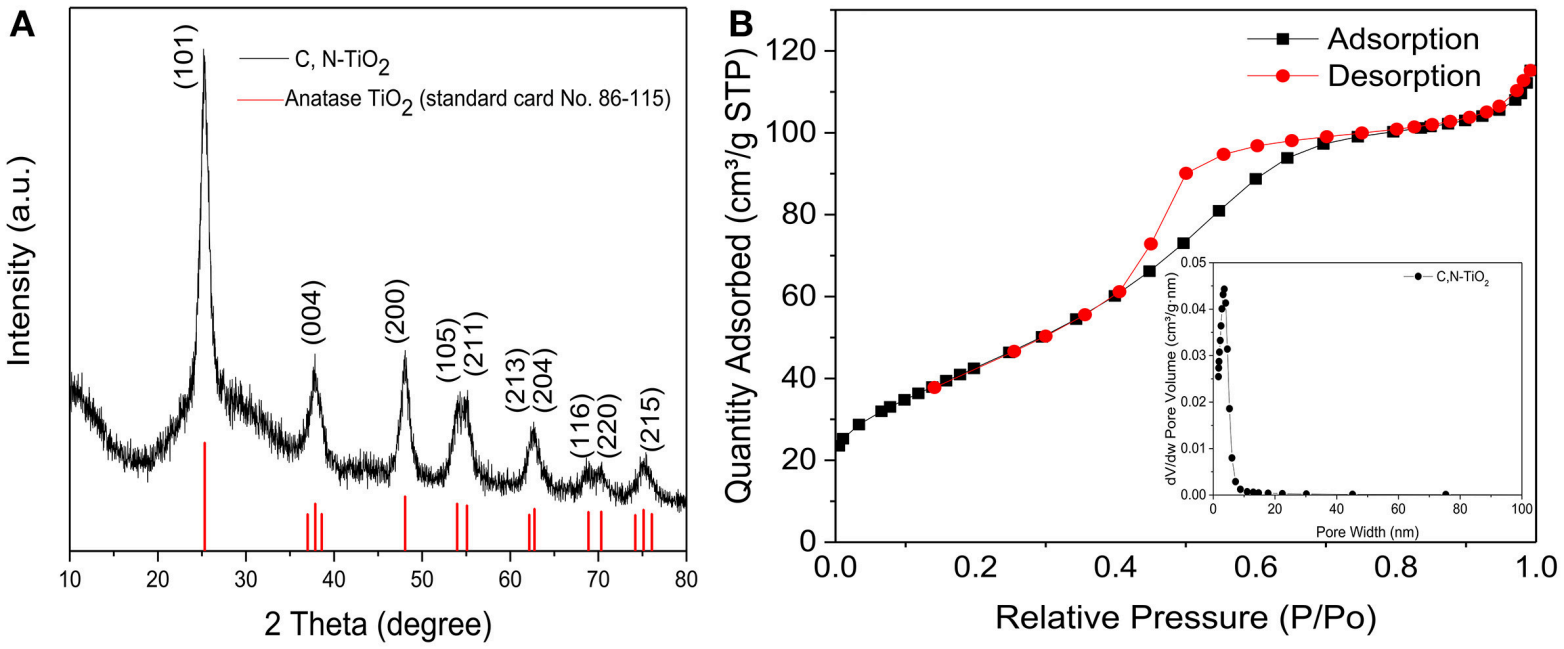
**(A)** X-ray diffraction (XRD) patterns of C, N-TiO_2_. **(B)** Nitrogen adsorption-desorption isotherms and the corresponding pore-size distributions (the inset) of C, N-TiO_2_.

As expected, the porous structure showed a large surface area of 159.903 m^2^/g and an average pore diameter of 3.64 nm (Figure 3B). As previously mentioned, since the specific surface area (SSA) is one of the key factors determining the catalytic efficiency of catalysts, it is reasonable to expect that the as-synthesized porous C, N-TiO_2_ would achieve enhanced catalytic performance in the following degradation of 4-nitrophenol compared to A-TiO_2_, with the grain size of 20 nm and surface area of 50 m^2^/g.

The crystal structures and particle size of the C, N-TiO_2_ were shown in Figure 3A. Only the anatase phase was detected from the diffraction peaks of the as-synthesized C, N-TiO_2_. This could be attributed to the alkaline reaction conditions during the synthesis process and the N-doping as well. It has been reported that carbon introduction does not modify the crystal of the TiO_2_ (Trevisan et al., 2014).

### Photocatalytic activity of C, N-TiO_2_

**Figure 4:**
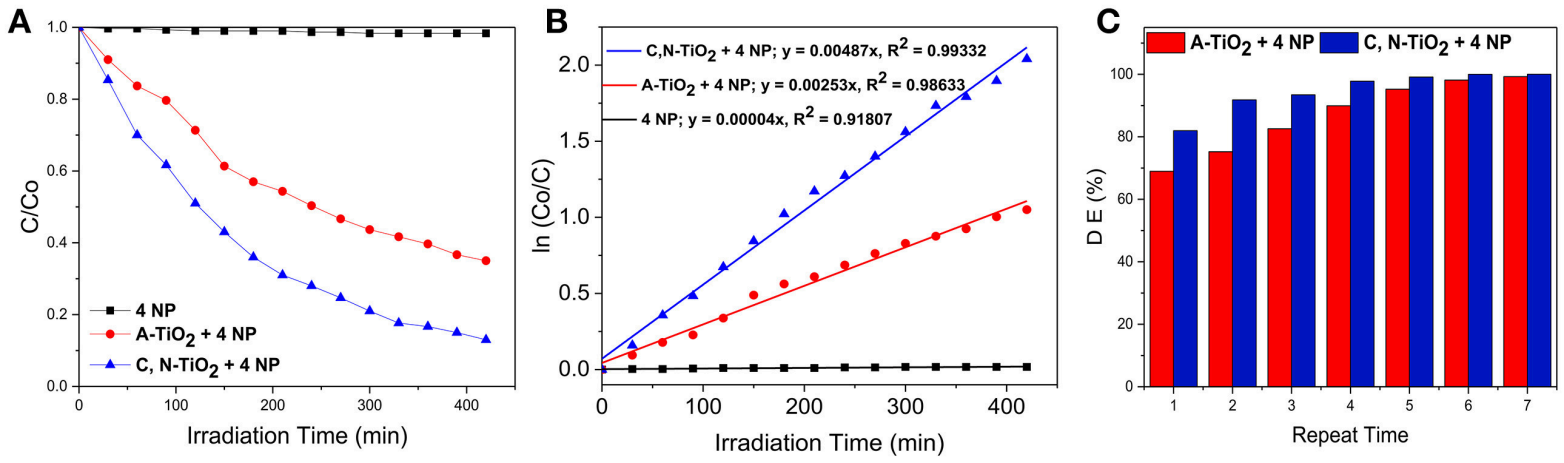
**(A)** Photocatalytic degradation of 4-nitrophenol. **(B)** First order kinetic analysis for the 4-nitrophenol degradation over C, N-TiO_2_ and A-TiO_2._ **(C)** Degradation of 4-nitrophenol over A-TiO_2_ and C, N- TiO_2_ after multiple runs.

The photocatalytic activity of C, N-TiO_2_ and A-TiO_2_ in the degradation of 4-nitrophenol from an initial concentration of 7.0 × 10^−2^ mM under simulated sunlight is shown in Figure 4A. The reactivity of the photocatalysts was represented by the ratio of residual concentration to initial concentration of 4-nitrophenol, C/Co, as a function of irradiation time. C, N-TiO_2_ and A-TiO_2_ degraded 87 and 65% of 4-nitrophenol in 420 min under simulated sunlight irradiation, respectively. The semi-logarithmic plots of concentration data gave a straight line (Figure 4B), indicating that the photocatalytic degradation of 4-nitrophenol can be described by the first-order kinetic model, ln *C* = –*kt* + ln *C*o, with a *k* constant of 4.87 × 10^−3^ min^−1^ and 2.53 × 10^−3^ for C, N-TiO_2_ and A-TiO_2_, respectively.

**Figure 5:**
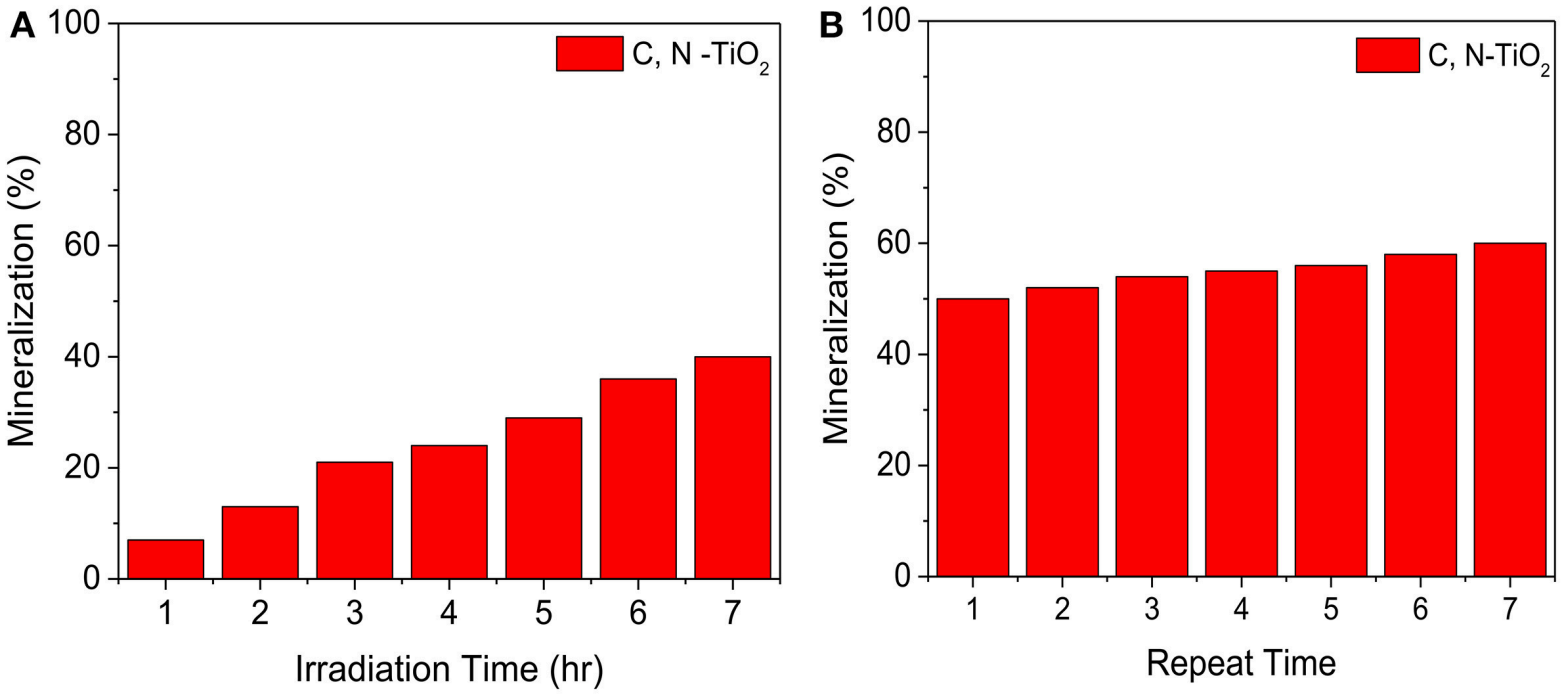
**(A)** TOC after 7 h degradation **(B)** TOC after multiple degradation.

However, a complete degradation was not achieved under 420 min irradiation: a challenge reported in several other studies (San et al., 2002; Hassan et al., 2014; Dong et al., 2015; Rezaei-Vahidian et al., 2017). To achieve a complete degradation, we repeated the photocatalytic degradation for multiple runs, under the same condition with freshly prepared catalysts at each repetition. A-TiO_2_ was able to achieve almost 100% degradation after 7 times of multiple treatments, while the C, N-TiO_2_ demonstrated a higher efficiency and achieved almost complete degradation on the 5th treatment (as shown in Figure 4C). Furthermore, according to the total organic carbon quantification, the photocatalytic degradation using C, N-TiO_2_ was able to mineralize the 4-nitrophenol to about 40% after 7 h (Figure 5A). And after 7 repeated treatments, 4-nitrophenol was mineralized to about 60% (Figure 5B).

### Zebrafish embryo toxicity test

**Figure 6:**
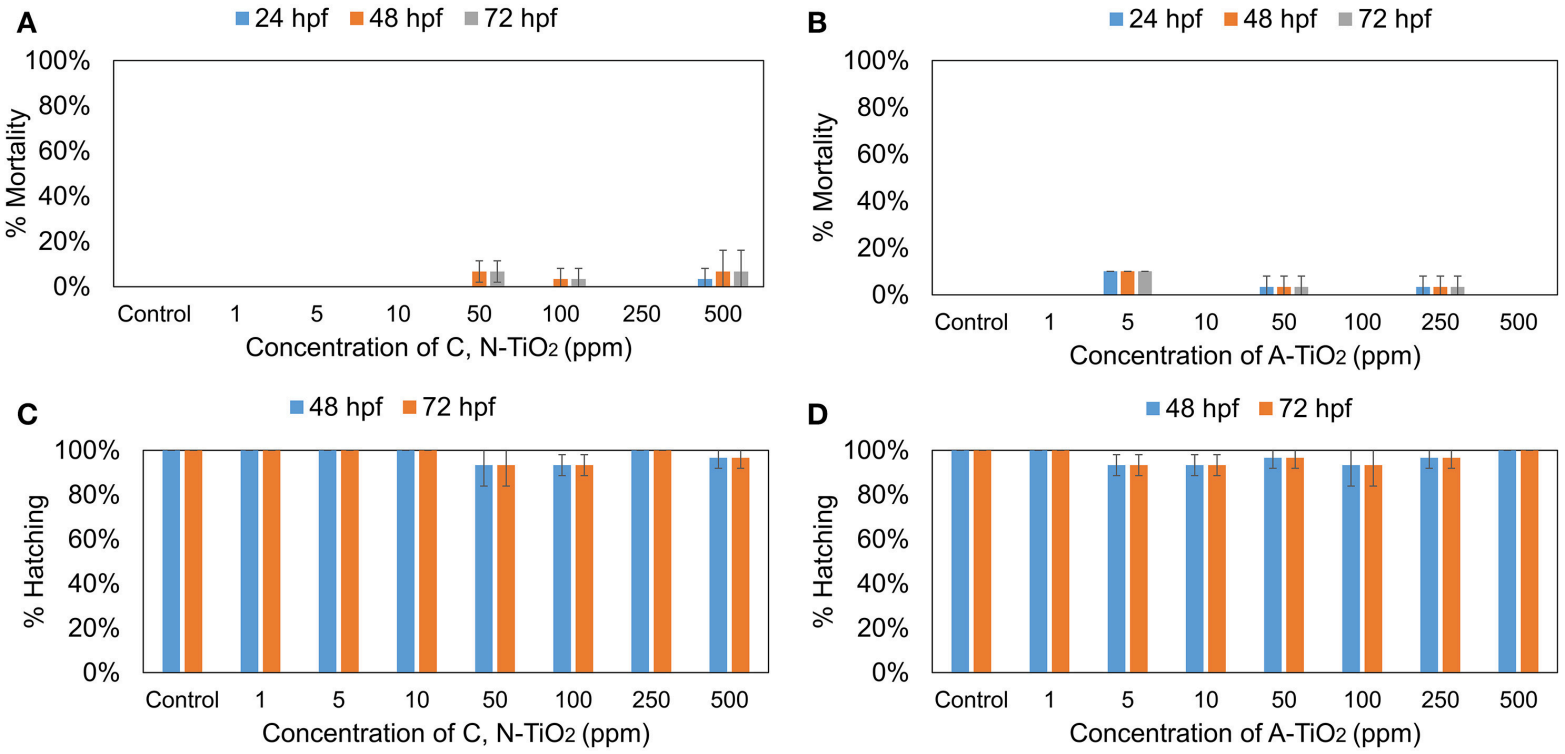
**(A,B)** Mortality rate and **(C,D)** Hatching rate of zebrafish treated with C, N-TiO_2_ and A-TiO_2_. (Values are expressed as means ± S.D).

To assess the toxic effects of 4-nitrophenol on zebrafish, embryos were treated with different concentrations (1, 5, 10, 15, and 20 mg/L) for 72 h. The hatching and mortality rates were assessed during the exposure period, at 24, 48, 72 hpf. Figure S5 shows that 4-nitrophenol exhibited a concentration-dependent toxicity, exerting a decreased hatching rates and increased mortality. To assess and compare the possible effects of C, N-TiO_2_ and A-TiO_2_, embryos were treated with different concentrations (1, 5, 10, 50, 100, 250, and 500 mg/L) of these samples for 72 h. Figure 6 shows that no obvious toxicity were found for both C, N-TiO_2_, and A-TiO_2_ at all concentrations tested. These results coincided well with Zhu et al. who found both nano and bulk forms of TiO_2_ were non-toxic to zebrafish (Zhu et al., 2008).

**Figure 7:**
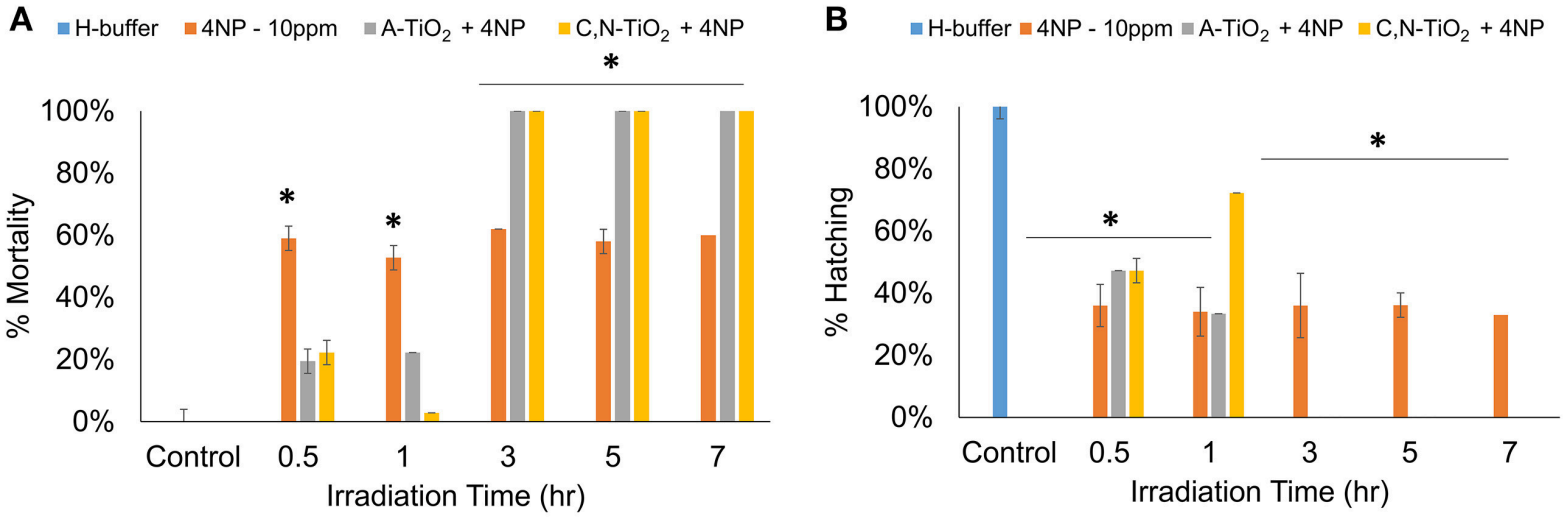
Degradation time-dependent toxicity of zebrafish treated after 72hpf. **(A)** Mortality rate, **(B)** Hatching rate. (Values are expressed as means ± S.D, ^*^P < 0.01).

Interestingly, the analysis on the intermediate compounds produced by photocatalytic degradation showed much higher toxicity than the parent compound. Embryos treated with intermediate compounds obtained within 1 h of degradation showed a decrease in toxicity as compared to 4-nitrophenol, exhibiting a decrease in mortality rates and an increase in hatching rates of zebrafish embryos. This effect could be attributed to a decrease in concentration of 4-nitrophenol during the photocatalytic process. However, highly significant effects were observed when embryos were exposed to products obtained after 3 h of degradation, possibly due to the formation of more toxic intermediate compounds. Results showed an increase in mortality rate and decrease in hatching rate in embryos treated with degradation products obtained after 3 h irradiation time at all developmental stages (Figure 7 and Figure S6). In particular, exposure to all intermediate products obtained after 3 h degradation led to 0% hatching rate and 100% mortality rate, respectively (as shown in Figure 7).

Comparative experiments showed that the hatching rate and mortality rate of embryos exposed to degradation products by A-TiO_2_ was significantly lower than that C, N-TiO_2_, suggesting that higher degradation efficiency led to the formation of more toxic intermediate compounds, thus causing more severe inhibition of hatch and a higher mortality rate. Figure S7 shows embryos treated with the degradation by-products obtained at 1, 3, and 7 h irradiation time after 72 hpf. We observed five types of malformations: pericardial edema (PE), tail malfunction (TM), bent spine (BS), unhatched dead embryo (UDE) and disintegrated embryo (DE). All samples induced high percentages of these abnormalities and malformations. However, malfunctions including unhatched dead embryo and disintegrated embryo were induced by byproducts obtained at 3 and 7 h irradiation time respectively.

**Figure 8:**
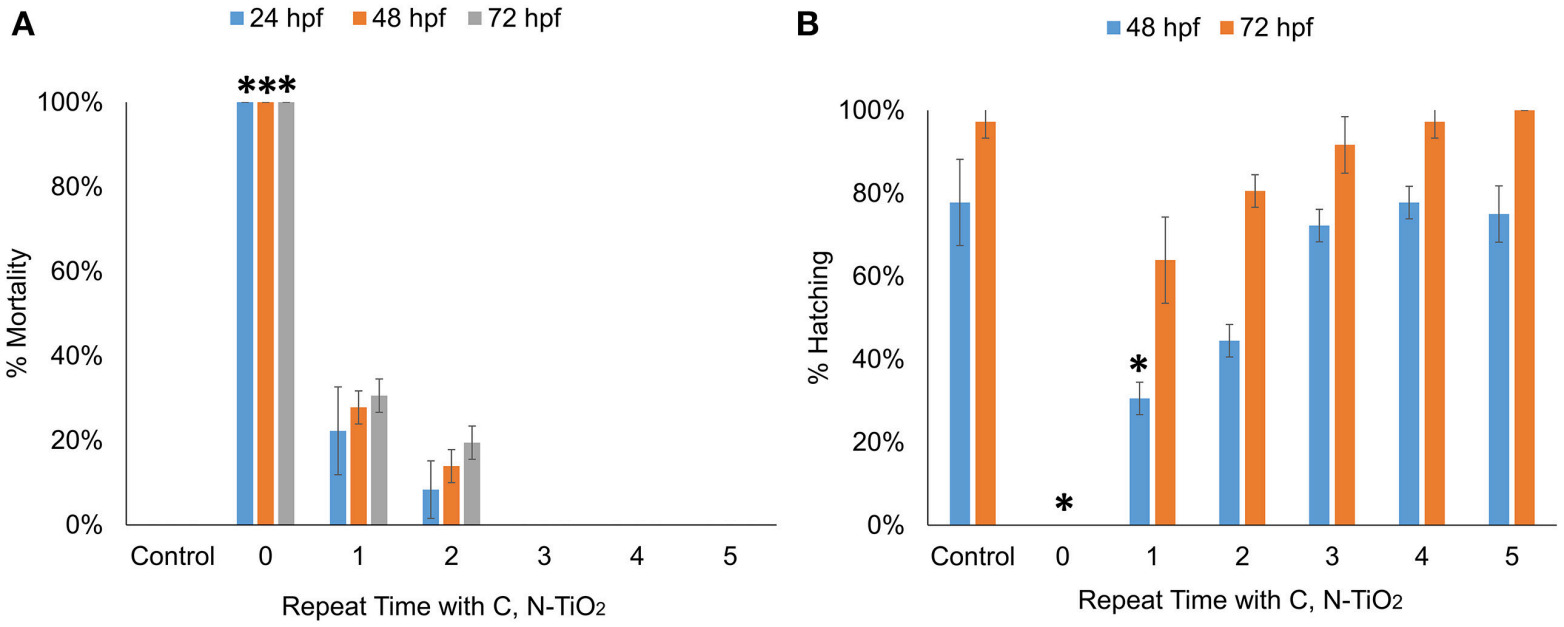
Multiple degradation time-dependent toxicity of zebrafish embryos after 24, 48, and 72 hpf. **(A)** Mortality rate **(B)** Hatching rate. (Values are expressed as means ± S.D, ^*^P < 0.01).

**Figure 9:**
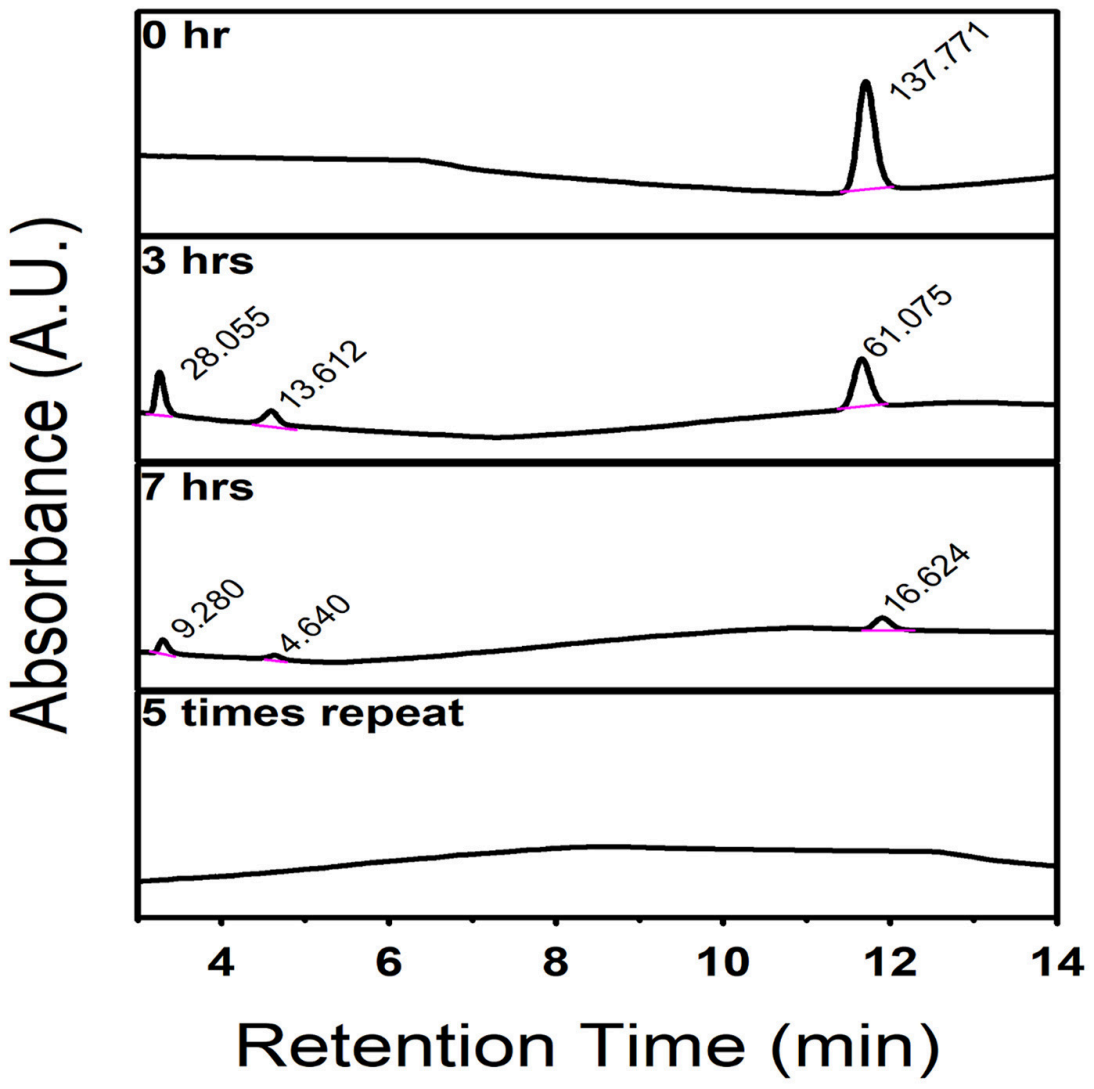
HPLC spectrum showing the degradation of 4-nitrophenol by C, N-TiO_2_, the intermediate compounds formation after 3 h irradiation and the complete degradation after 5 repeated process.

The mortality rates and hatching rates of zebrafish embryos exposed to intermediate products obtained after multiple degradation at 24, 48, and 72 hpf are shown in Figures 8A,B. There was a significant decrease in toxicity after the first run of degradation. Exposure to products obtained after multiple degradation had no significant effect on the mortality rate and hatching rate of embryos, suggesting that all toxic intermediate compounds had been successfully degraded. HPLC analyses were consistent with this statement. As Figure 9 showed, before degradation, 4-nitrophenol showed a distinct peak at the mark of 12-min elution time. After 3 h irradiation, a decreased of the peak at 12-min mark indicated a decrease in the concentration of 4-nitrophenol while the appearance of two small peaks at the marks of 3 and 5-min indicated the formation of intermediate compounds. With 5 repeated treatment, all peaks disappeared indicated a complete degradation of 4-nitrophenol therefore no toxicity was observed.

According to our HPLC study shown in Figure S8 as well as the analysis result reported by Luo et al. (2013), the intermediate compounds (represented by the peaks appear in the 3-min and 5-min elution time) are likely 4-nitrobenzene-1,2-diol and 4-nitrobenzene-1,3-diol. The compounds were likely the same composition but different concentrations when using A-TiO_2_ and C, N-TiO_2_ as the catalyst respectively.

## Conclusion

In summary, through C and N co-doping, we have synthesized a type of mesoporous C, N-TiO_2_ photocatalyst by a simple sol-gel method combined with calcination process. The modified photocatalyst exhibited a large surface area, pore volume and simple crystal structure of anatase. C, N-TiO_2_ showed higher photocatalytic efficiency during the degradation of 4-nitrophenol than pure A-TiO_2_ under simulated light irradiation due to the synergistic effect of carbon and nitrogen co-doping. However, a complete degradation was not achieved under 420 min and intermediate degradation products of 4-nitrophenol displayed a much higher toxicity in zebrafish embryos. A repeated degradation process was necessary to achieve complete degradation and render the compound non-toxic to the zebrafish embryos. Our results further emphasized the necessity of paying close attention to the toxicity potentials of degradation compounds while performing photocatalytic degradation to ensure comprehensive assessment of the threats and significance of these chemicals to the natural environment.

## Author contributions

OO contributed to the design and planning of the work, performed experiments, data analysis and drafted the manuscript. TY contributed in the zebrafish embryonic toxicity assessment and graphical design. XCa contributed to the HPLC analysis. YJ contributed to the photodegradation experiments. XCh contributed to the materials synthesis and data analysis. GP contributed to the toxicity assessment and data analysis. RL, YQ, and SL designed, planned and supervised the research and contributed to the manuscript writing. All authors read and approved the final manuscript.

### Conflict of interest statement

The authors declare that the research was conducted in the absence of any commercial or financial relationships that could be construed as a potential conflict of interest.
