# Supplementary material for: Photocatalytic Degradation of 4-Nitrophenol by C, N-TiO2: Degradation Efficiency vs. Embryonic Toxicity of the Resulting Compounds
Source: Front Chem. 2018 Jun 4;6:192. doi: 10.3389/fchem.2018.00192 (PMC5994427; doi:10.3389/fchem.2018.00192)
Supplement: Supplementary file 1 [file Data_Sheet_1.docx]

Supplemental Information

**Photocatalytic Degradation of 4-Nitrophenol by C, N-TiO_2_: Degradation Efficiency *vs.* Embryonic Toxicity of the Resulting Compounds**

**Oluwatomiwa A. Osin^1,2^, Tianyu Yu^1,2^, Xiaoming Cai^3^, Yue Jiang^1^, Guotao Peng^1^, Xiaomei Cheng^5^, Ruibin Li^4^, Yao Qin^5,*^, Sijie Lin^1,2,*^**

^1^College of Environmental Science and Engineering, State Key Laboratory of Pollution Control and Resource Reuse, Shanghai Institute of Pollution Control and Ecological Security, Biomedical Multidisciplinary Innovation Research Institute, Shanghai East Hospital, Tongji University, Shanghai 200092, China;

^2^UN Environment-Tongji Institute of Environment for Sustainable Development, Tongji University, Shanghai 200092, China;

^3^Center for Genetic Epidemiology and Genomics, School of Public Health, Jiangsu Key Laboratory of Preventive and Translational Medicine for Geriatric Diseases, Medical College of Soochow University, Suzhou, Jiangsu 215123, China;

^4^School for Radiological and Interdisciplinary Sciences (RAD-X), Jiangsu Provincial Key Laboratory of Radiation Medicine and Protection, Medical College of Soochow University, Suzhou, Jiangsu 215123, China;

^5^**The Institute for Translational Nanomedicine, Shanghai East Hospital, The Institute for Biomedical Engineering & Nano Science, Tongji University School of Medicine, Shanghai 200092, China**

*** Correspondence:**

Sijie Lin, Ph.D. Professor

College of Environmental Science and Engineering, Tongji University

1239 Siping Road, Shanghai 200092, China

Tel: 86 21 65982325

E-mail: [lin.sijie@tongji.edu.cn](mailto:lin.sijie@tongji.edu.cn)

Yao Qin, Ph.D. Associate Professor

**Tongji University School of Medicine,** Tongji University

E-mail: lilyqin@tongji.edu.cn

##
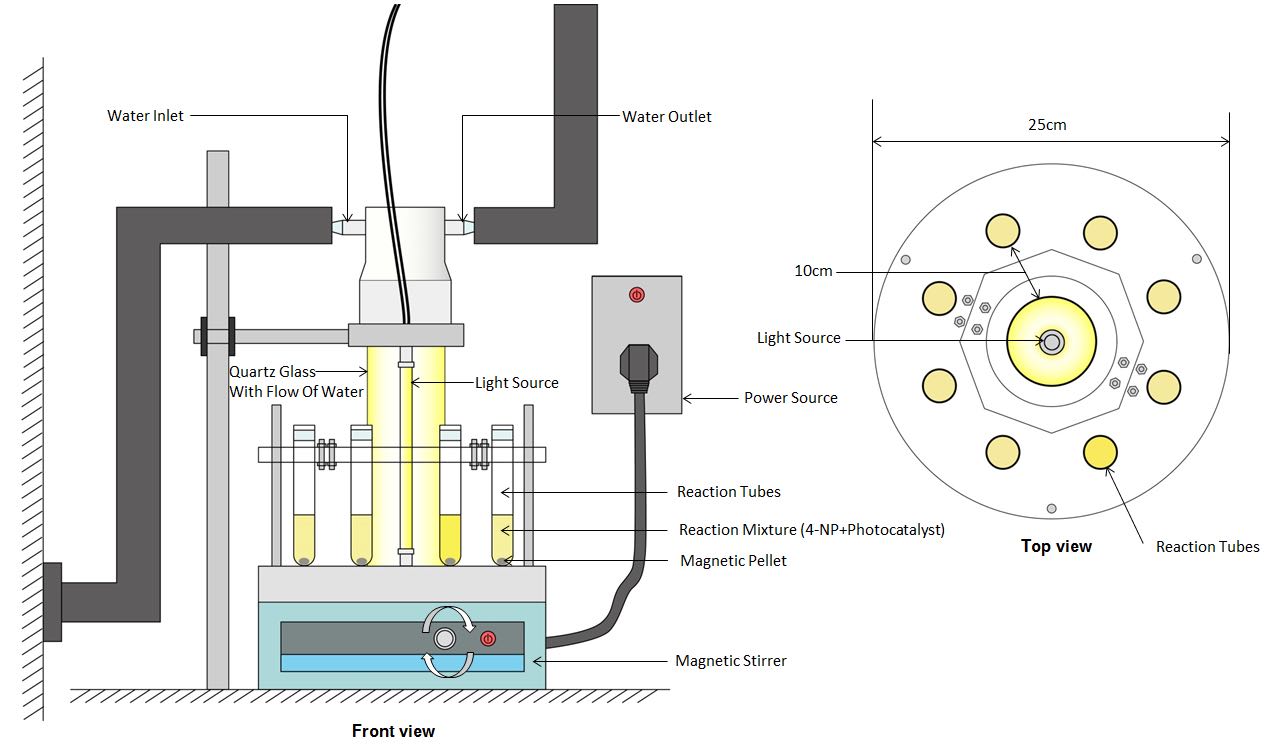


## Figure S1. Experimental setup of photoreactor used for photocatalytic degradation of 4-nitrophenol. Figure S2. High resolution XPS spectra of C, N-TiO_2_: O1s.


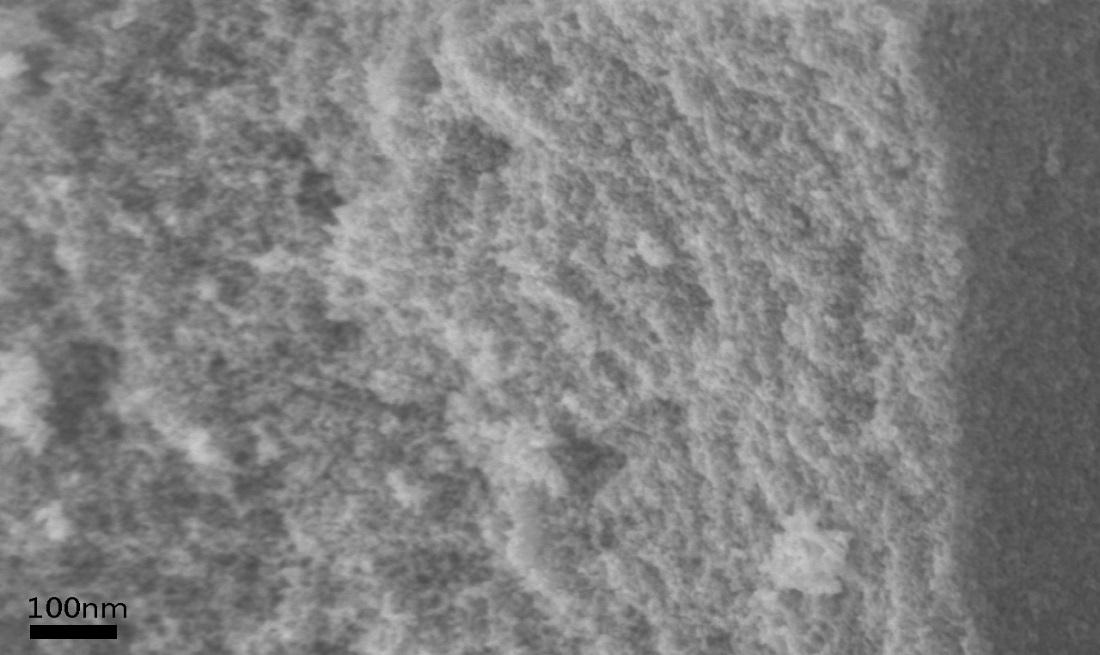


**Figure S3.** Low resolution SEM image of C, N-TiO_2_.

**
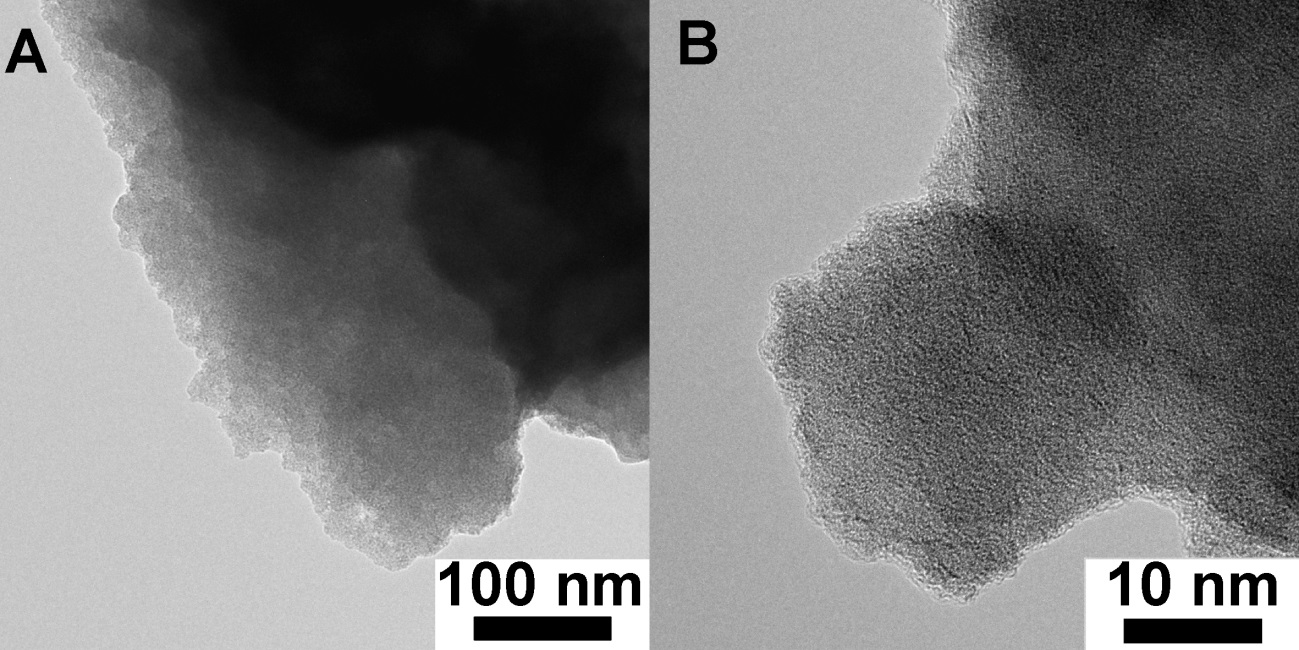
**

**Figure S4** TEM images of C, N-TiO_2_ without addition of perchloric acid.


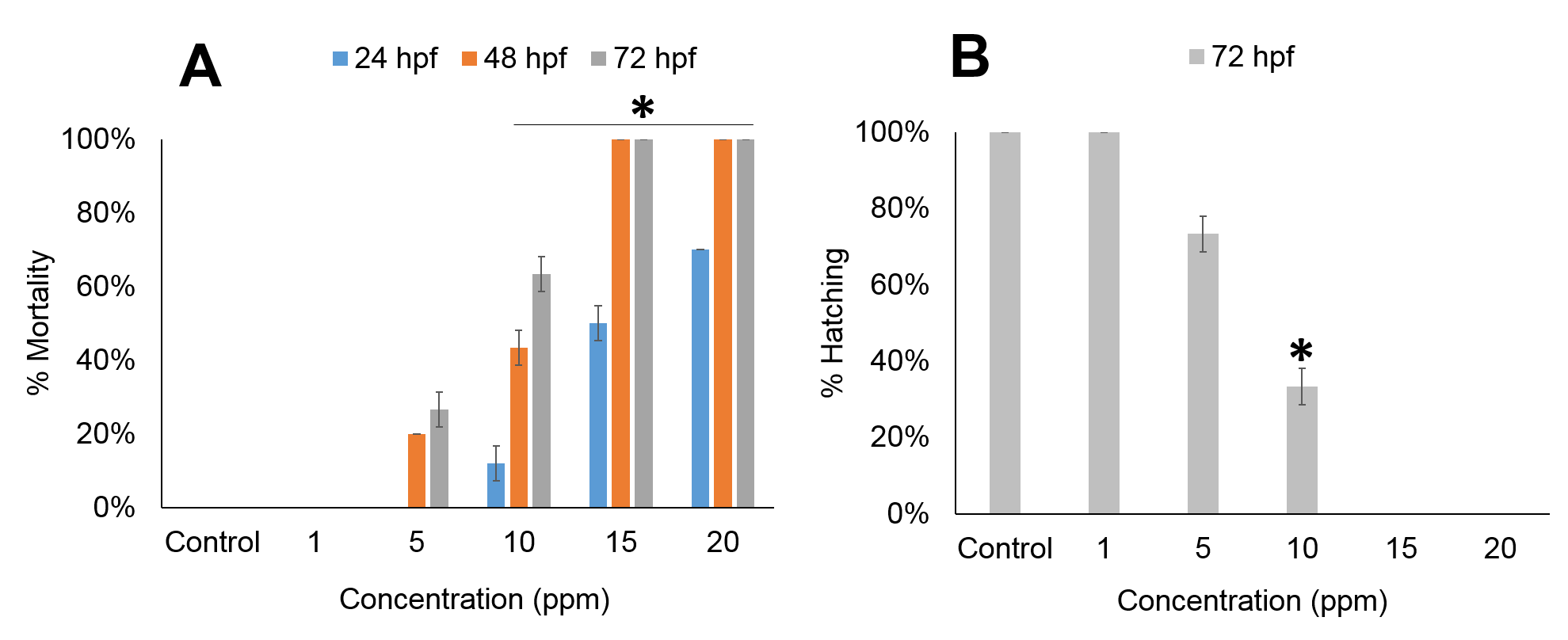


**Figure S5. Toxicity profile of 4-nitrophenol in zebrafish embryos. A.** Concentration dependent mortality exerted by 4-nitrophenol at 24, 48 and 72 hpf; **B.** Concentration dependent hatching percentage of zebrafish embryos upon 4-nitrophenol exposure at 72 hpf**.** Values are expressed as means **±** S.D, * = P < 0.01).

**
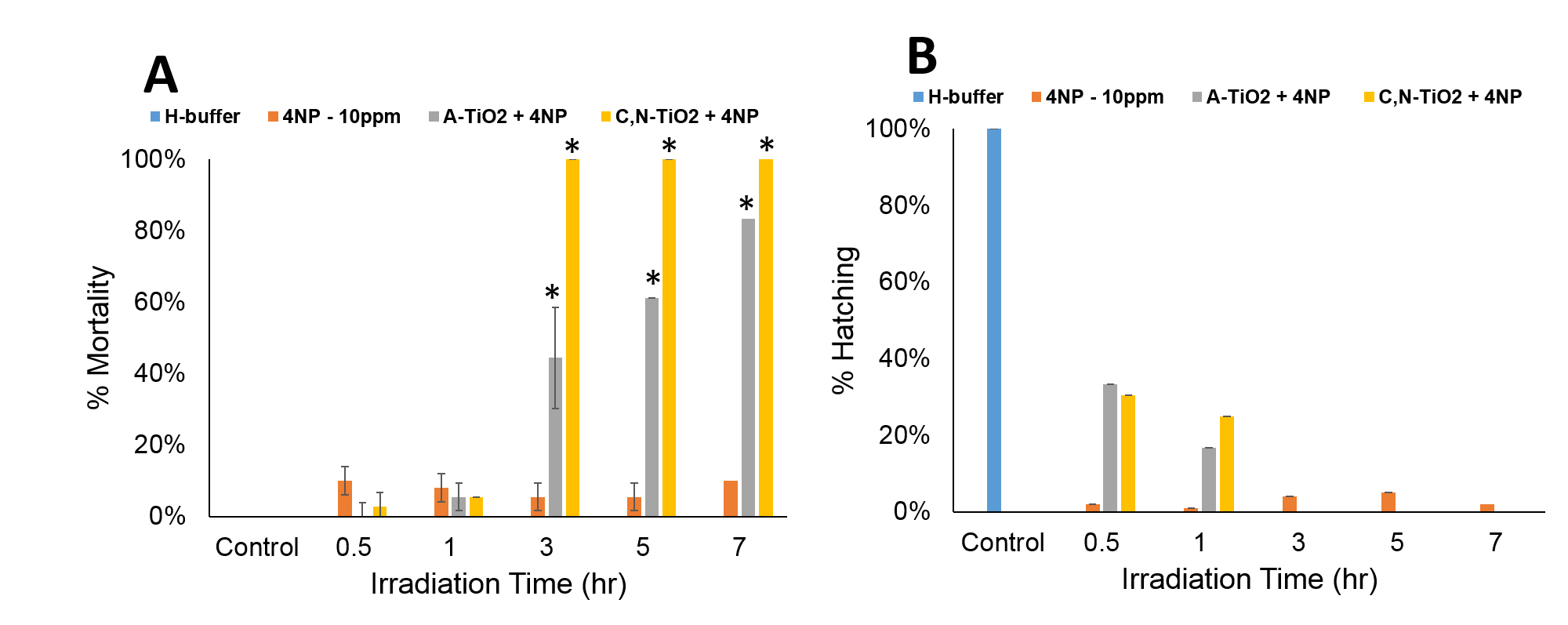
**

**Figure S6.** Toxicity profile of 4-nitrophenol and its degradation compounds resulted from different irradiation time. A. Mortality of zebrafish embryos at 24 hpf; B. Hatching percentage of zebrafish embryos at 48 hpf. Values are expressed as means ± S.D, * = P < 0.01.


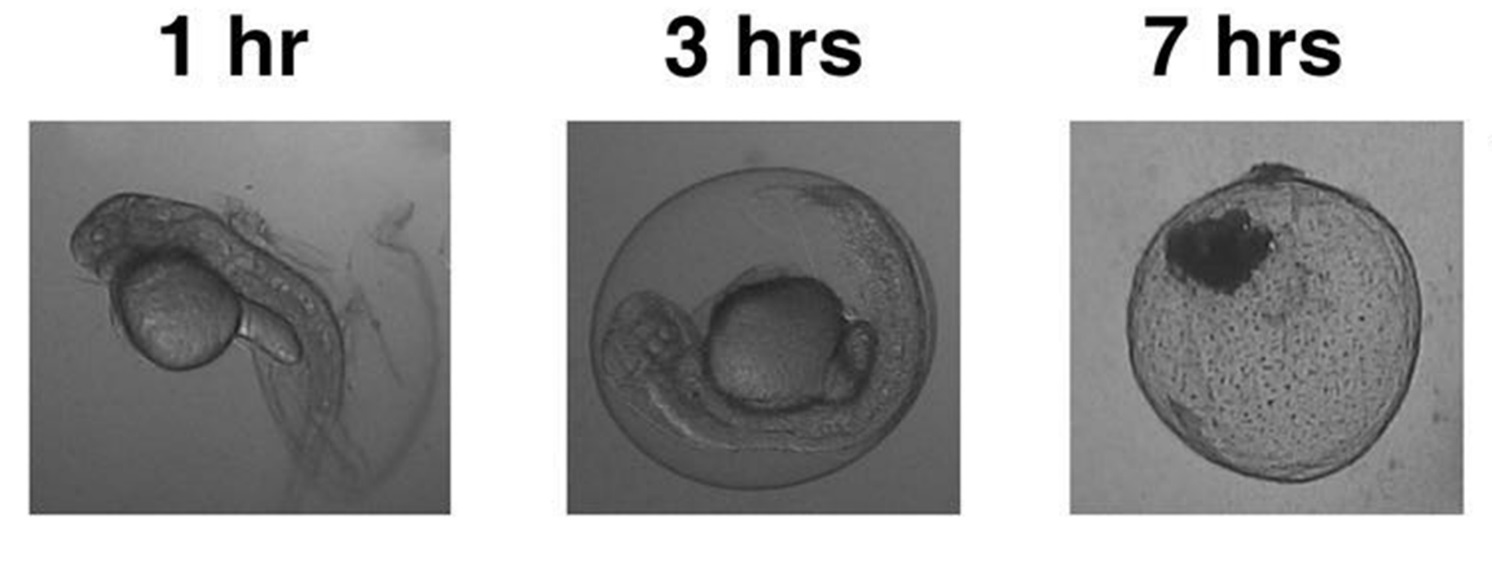


fdtrhtrhrer

**Figure S7.** Representative microscopic images of zebrafish embryos (72 hpf) exposed to the degradation compounds of 4-nitrophenol after different irradiation time.


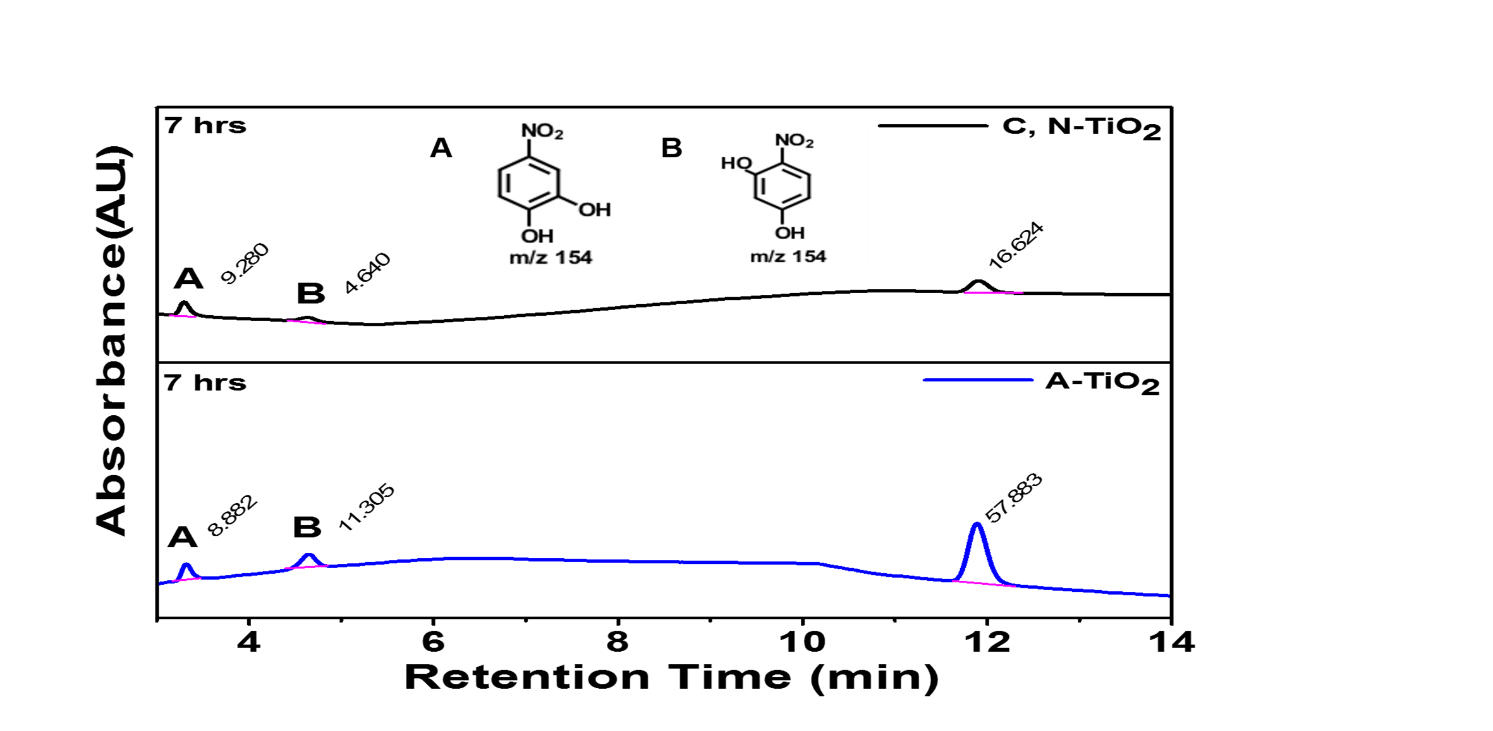


**Figure S8.** HPLC spectrum showing the intermediate products formed after 7 hours degradation of 4NP over C, N-TiO_2_ and A-TiO_2_
